# Benchmark Investigation of Band-Gap Tunability of Monolayer Semiconductors under Hydrostatic Pressure with Focus-On Antimony

**DOI:** 10.3390/nano10112154

**Published:** 2020-10-29

**Authors:** Xiangyu Dai, Zhengfang Qian, Qiaolu Lin, Le Chen, Renheng Wang, Yiling Sun

**Affiliations:** 1 Key Laboratory of Optoelectronic Devices and Systems of Ministry of Education and Guangdong Province, College of Physics and Optoelectronic Engineering, Shenzhen University, Shenzhen 518060, China; daixiangyu@szu.edu.cn (X.D.); linql15@lzu.edu.cn (Q.L.); wangrh@szu.edu.cn (R.W.); sunyl@szu.edu.cn (Y.S.); 2 School of Physics and Telecommunication Engineering, Guangxi Colleges and Universities Key Lab of Complex System Optimization and Big Data Processing, Yulin Normal University, Yulin 537400, China

**Keywords:** monolayer antimony, band-gap tunability, first principle, optoelectronics

## Abstract

In this paper, the band-gap tunability of three monolayer semiconductors under hydrostatic pressure was intensively investigated based on first-principle simulations with a focus on monolayer antimony (Sb) as a semiconductor nanomaterial. As the benchmark study, monolayer black phosphorus (BP) and monolayer molybdenum disulfide (MoS_2_) were also investigated for comparison. Our calculations showed that the band-gap tunability of the monolayer Sb was much more sensitive to hydrostatic pressure than that of the monolayer BP and MoS_2_. Furthermore, the monolayer Sb was predicted to change from an indirect band-gap semiconductor to a conductor and to transform into a double-layer nanostructure above a critical pressure value ranging from 3 to 5 GPa. This finding opens an opportunity for nanoelectronic, flexible electronics and optoelectronic devices as well as sensors with the capabilities of deep band-gap tunability and semiconductor-to-metal transition by applying mechanical pressure.

## 1. Introduction

Recently, two-dimensional (2D) nanomaterials have exceptional properties for optoelectronic nanodevices [1,2,3,4,5]. Among them, graphene has attracted a lot of attention, with limited device applications in electronics and optoelectronics due to its zero band-gap [6,7,8].

Besides graphene, atomically thin 2D forms of layered transition metal dichalcogenides (TMDs) have recently attracted remarkable scientific and technical interest [9,10,11,12,13,14] due to their wide range of band-gaps. Among various TMDs, molybdenum disulfide (MoS_2_) has been the most extensively studied in 2D forms because of its stability at room temperature in air [10], alternative preparation methods [14,15,16], and outstanding electronic properties [17,18]. In contrast, black phosphorus (BP), a kind of bulk counterpart of phosphorus with a layered structure, has also been widely investigated [19,20,21,22]. As reported, monolayer antimony (Sb), i.e., antimonene, was synthesized on germanium in 2017 [23]. It is a semiconductor with a wide band-gap and special physical properties [24]. It should be emphasized that Sb-based compounds are also interesting for optoelectronics, such as Sb halide perovskites and their derivatives [25].

There are many interesting layer-dependent properties in 2D nanomaterials [17,18,19], which differ greatly from the properties of their bulk materials. Especially, the indirect band-gap of 1.3 eV for bulk MoS_2_ increases to a direct band-gap of 1.8 eV in single-layer form. Moreover, monolayer BP shows great mobility and in-plane anisotropy, which makes BP a potential material in the optoelectronic and energy device field. In contrast, antimony (Sb) in bulk is a typical metalloid element with metallic luster. Therefore, single-layer semiconductors as novel nanomaterials deserve to be deeply investigated, especially for novel electronic and optoelectronic device applications.

Recently, the band-gap engineering of 2D semiconductors has attracted more research interest by means of chemical functionalization [26,27,28], in-plane strain [29,30,31,32], or by applying an external electric field. However, the band-gap engineering for monolayer Sb under hydrostatic pressure has not been investigated so far. The hydrostatic pressure can isotropically provide both in-plane and out-of-plane stresses to single-layer structures, which can affect the chemical bond of monolayer semiconductors. Applying hydrostatic pressure has been proven effective to tune the electronic structures of 2D semiconductors [33].

This paper investigated the band-gaps of three single-layer semiconductors, i.e., MoS_2_, BP, and Sb, based on first-principle calculations, focusing on the band-gap tunability of single-layer semiconductors under hydrostatic pressure. In this paper, the band-gap changes of the monolayer semiconductors of Sb, BP, and MoS_2_ under hydrostatic pressure were systematically investigated for exploring the band-gap tunability. We found that the band-gap value of the monolayer Sb could be well controlled by applying hydrostatic pressure. Its pressure sensitivity and band-gap tunability were more effective than that of the monolayer MoS_2_ and BP. Moreover, the electrical structures of the monolayer Sb were also studied under different hydrostatic pressures. The breakage of bonds and the phase transition were predicted to occur above a critical hydrostatic pressure ranging from 3 to 5 GPa. This finding opens a new opportunity for device applications of monolayer Sb to nanoelectronics, MEMS, and sensors.

## 2. Materials and Methods

**Figure 1:**
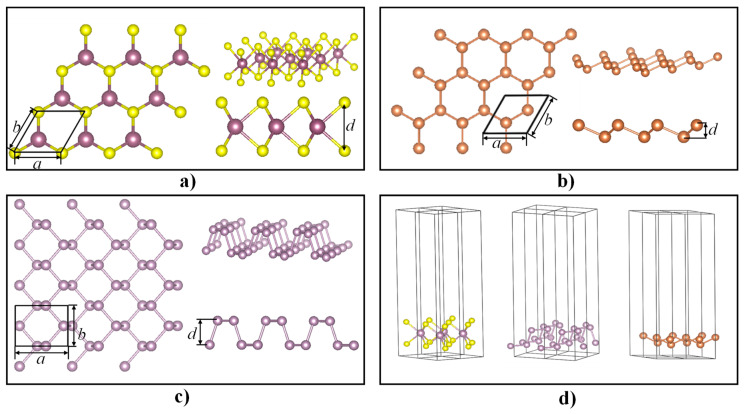
Schematic illustrations of atomic models: (**a**) monolayer molybdenum disulfide (MoS_2_), (**b**) monolayer black phosphorus (BP), (**c**) monolayer antimony (Sb), and (**d**) computational models with the vacuum layer.

The atomic structure of monolayer Sb is similar to that of monolayer MoS_2_ and BP with buckled honeycombs under ambient conditions [34,35]. The atomic models of the monolayer MoS_2_, BP, and Sb are shown in Figure 1a–c, respectively. The parameters of a and b represent the in-plane lattice parameters of the monolayer semiconductors, while the parameter of d represents the out-of-plane lattice parameter, i.e., the thickness of the monolayer structures. The atomic models and lattice parameters of BP and MoS_2_ were provided by the Cambridge Crystallographic Data Center (CCDC). The lattice parameters of the Sb monolayer were calculated, and a = b = 4.141 Å and d = 3.0 Å were obtained after relaxation. When performing first-principle simulations for monolayer materials, a vacuum layer in the z-direction between two monolayers is commonly used for isolating self-interaction and interlayer effects. A vacuum layer thickness ranging from 15 to 20 Å is usually adopted in the literature [33,36].

In this benchmark study, all first-principle calculations were carried out by both the Vienna Abinitio Simulation Package (VASP, version: 5.4.4, developers: Hafner Research Group, location of the manufacturer: University of Vienna, Vienna.) [37] and the Cambridge Sequential Total Energy Package (CASTEP, version: 7.0, name of developers: Accelrys, now BIOVIA, location of the manufacturer: San Diego, California.) based on density functional theory (DFT) for comparison. There is no difference between CASTEP and VASP in the description of electron interaction; however, they build the atomic structures by applying pressure in different ways. The hydrostatic pressure is isotropically applied in 3D. In VASP, the target pressure is applied to the primary diagonals of the residual stress matrix of the atomic structure. It is then processed by optimizing the atomic structure until the residual stress components are decreased to zero. Alternatively, in CASTEP, the x, y, and z coordinates of each atom are scaled by the factor that is determined by applied pressure. Moreover, the pressure on the atomic structure is calculated and updated at each ion iteration step according to the diagonals of the residual stress matrix.

Furthermore, in the VASP simulation, the ultrasoft pseudopotential (USPP) is applied and the generalized gradient approximation (GGA) with the parameterized Perdew–Burke–Ernzerh (PBE) functional is adopted as the exchange correlation function. For the USPP, to reduce the plane wave basis set and cut-off radius, the closely interacting electrons in the atomic core region are removed by generalized orthogonal conditions. Because only the close interaction electron orbitals in the atomic core region need high cut-off energy, with less sacrifice of calculation accuracy, the calculation efficiency is greatly improved when the USPP is used, and it is easier to find geometric stable states under pressure conditions. Kohn–Sham schemes based on GGA functionals can describe the total energy and related properties of crystals and molecules accurately but underestimate the band-gap. In CASTEP, GGA with Heyd–Scuseria–Ernzerh screened hybrid functional (HSE06) is applied as the exchange correlation function, which includes an empirical scissors correction based on a screened Coulomb potential [38]. The empirical scissors correction is effectively a rigid shift of the conduction band with respect to the valence band in semiconductors and insulators and can expand the underestimated band-gap calculated by the GGA functional [39,40]. However, the correction value of the scissors operator to the energy band is inversely proportional to the square of the charge distance, so when the structure is deformed under pressure, the correction value of the scissors operator fluctuates, resulting in a change in the shape of the energy band.

As explained in above section, there are large differences of the PBE and HSE06 calculated band-gaps reported in the literature [39,40]. Therefore, we simultaneously performed and compared the results using the VASP and CASTEP packages with PBE and HSE06 functionals, respectively, to validate the results under hydrostatic pressure and to obtain more reliable data as well as a range of band-gap tunability.

Moreover, in the simulation model, the plane wave energy cut-off was set to 550 eV and the K-space was divided by a 15 × 15 × 1 mesh along the directions of a, b, and c, respectively. The conjugate gradient algorithm was used for ion iteration and ion coordinates during the process of geometry optimization. The cell volume and cell size were not constrained.

## 3. Results

### 3.1. Benchmark Study of the Band-Gap Calculations of the Three Monolayer Semiconductors

Band-gap is a fundamental physical parameter for semiconductors, which is not only related to bonding properties and crystal structures, but also to photon and electron transport properties.

A band-gap benchmark study of the monolayer Sb was performed using different Hamiltonians. The calculated results are listed in Table 1 for the comparison of the GGA combinations of the local density approximation (LDA), PBE, and HSE06 functionals. A band-gap of 1.21 was predicted by LDA, which is well known to underestimate the band-gap, and a band-gap of 1.27 eV was obtained using the GGA-PBE functional. A much higher value of 1.87 eV was obtained using GGA with the HSE06 functional by using VASP. However, it is still lower than the 2.28 eV that was obtained using CASTEP with the HSE06 functional [24]. We also verified that a very close band-gap value of 2.26 eV for the monolayer Sb was obtained by using the same package of CASTEP with the HSE06 functional. Based on the benchmark, VASP-GGA-PBE, VASP-GGA-HSE06, and CASTEP-GGA-HSE06 were used to perform DFT calculations for a comprehensive band-gap study of the three monolayer semiconductors, specifically the monolayers of Sb, BP, and MoS_2_ under hydrostatic pressure loadings.

VASP: Vienna Abinitio Simulation Package; CASTEP: Cambridge Sequential Total Energy Package; LDA: local density approximation; GGA: generalized gradient approximation; PBE: Perdew-Burke-Ernzerh functional; HSE06: Heyd-Scuseria-Ernzerh screened hybrid functionalFurthermore, the first-principle simulation results are listed in Table 2 for the monolayer BP and MoS_2_ with a comparison of the experimental (EXP) data available from literature [18,41]. As shown in Table 2, three monolayer semiconductors under investigation were consistently obtained using VASP-GGA with PBE, acting as a lower bound of band-gap values. Higher values were also consistently obtained by using CASTEP-GGA with HSE06, acting as the upper bound of band-gap values. The average values of VASP-GGA-PBE (V-P) and CASTEP-GGA-HSE06 (C-H) were then calculated as the average V-P/C-H. The relative deviation of the average V-P/C-H with experimental data from [18,41] was calculated as values of −5.59% and 4.26% for BP and MoS_2_, respectively. The deviation around 5% is reasonably close to the experimental results. At this time, the experimental data for the monolayer Sb are not available (NA). However, its band-gap can be predicted around the value of 1.775 eV based on our benchmark calculations. In contrast, the relative deviation of VASP-GGA-HSE06 (V-H) with the experimental data from [18,41] was calculated as relative values of 13.79% and 5.79% for BP and MoS_2_, respectively, which indicates the V-H can be a reasonable model for band-gap calculations.

**Figure 2:**
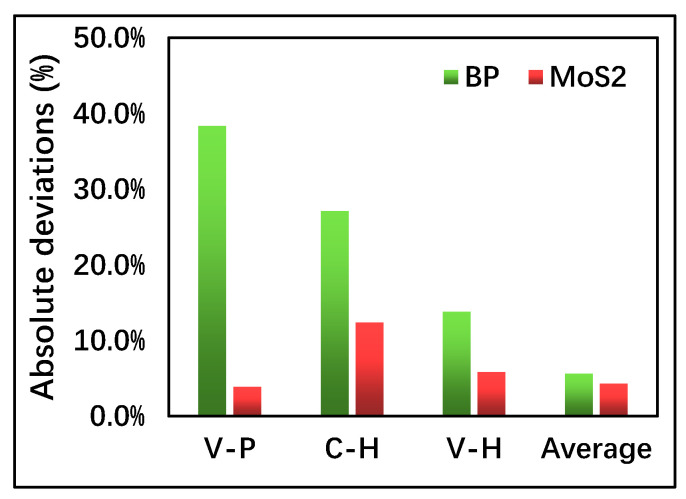
Statistics of the absolute deviations of the simulation results from the EXP data. V-P, C-H, and V-H represent the VASP-GGA-PBE, CASTEP-GGA-HSE06, and VASP-GGA-HSE06, respectively. “Average” represents the mean value of the V-P and C-H data.

Figure 2, furthermore, depicts the absolute (ABS) deviation percentage between the simulation results and the EXP data. Compared to VASP-GGA-PBE (V-P) and CASTEP-GGA-HSE06 (C-H), the results based onVASP-GGA-HSE06 (V-H) are closer to the EXP data. The comparison of the average V-P/C-H with the EXP data is surprisingly good, which suggests that the band-gap calculations are likely bounded by the V-P and C-H. Although the lower and upper bonds of the band-gap calculations of the monolayer semiconductors need further investigation from both theory and extensive calculations, in this paper, using both V-P and C-H for band-gap calculations can serve as a more reliable band-gap analysis and a range of band-gap tunability for benchmark investigation.

### 3.2. Band-Gap Tunability by Applying Hydrostatic Pressure

**Figure 3:**
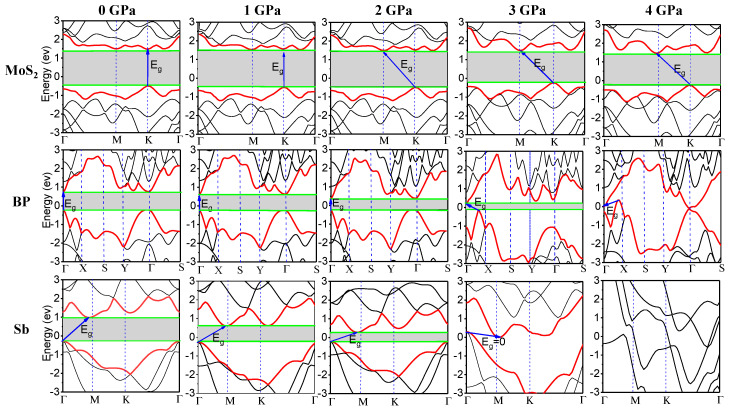
Evolution of the band structures of the monolayer semiconductors of MoS_2_, BP, and Sb under different hydrostatic pressures.

The band-gaps of the monolayer semiconductors of Sb, BP, and MoS_2_ were significantly changed by applying different values of hydrostatic pressure, based on our first-principle calculations. Figure 3 shows the evolution of the band structures of MoS_2_, BP, and Sb at 0, 1, 2, 3, and 4 GPa, respectively. Both MoS_2_ and BP show direct band-gaps, located at the high symmetry points K and Γ, respectively, at 0 GPa pressure. However, the monolayer Sb show indirect band-gaps. The global conduction band minimum (CBM) is located at points between the high symmetry points Γ and X, while the global valence band maximum (VBM) is located at the high symmetry point Γ. When the applied pressure was further increased to 2 GPa, the locations of the CBM of MoS_2_ and the VBM of BP changed. It is also shown that the band-gap eventually decreased to 0 eV at 4 GPa hydrostatic pressure for BP and Sb, but not MoS_2_, which requires much higher pressure for tuning the band-gap.

**Figure 4:**
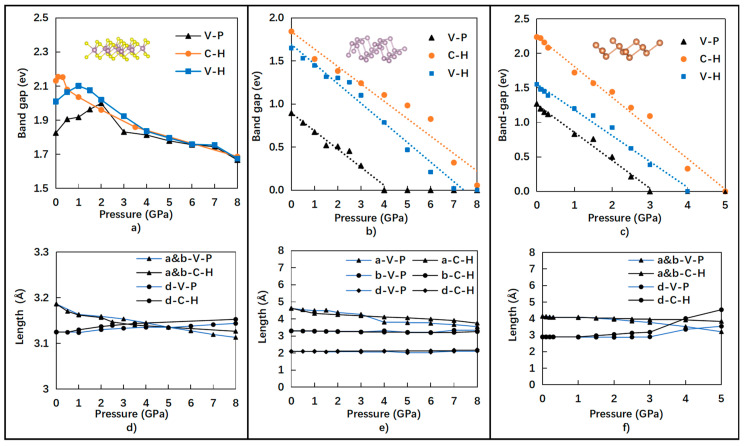
Relationships between band-gap (eV) and pressure (GPa) of (**a**) monolayer MoS_2_, (**b**) monolayer BP, and (**c**) monolayer Sb under different pressures, as well as the lattice parameters of (**d**) monolayer MoS_2_, (**e**) monolayer BP, and (**f**) monolayer Sb with applied pressure values.

Further investigation was performed by applying different pressures from 0 to 8 GPa for 12 case studies based on VASP-GGA-PBE (V-P), VASP-GGA-HSE06 (V-H), and CASTEP-GGA-HSE06 (C-H). The extensive calculation results are summarized in Figure 4a–c for the band-gap evolution of the monolayer semiconductors of Sb, BP, and MoS_2_ under different hydrostatic pressures. The values of band-gaps calculated by V-P and C-H under different pressures ranging from 0 to 8 GPa are consistently shown in Figure 4a–c as upper and lower bonds, respectively.

However, the band-gap value of the monolayer MoS_2_ did not change monotonically with applied pressures. When the applied pressure was increased, the direct band-gap initially increased, up to the maximum value. The responding pressure values, however, were dependent on the computational functionals, as shown in Figure 4a. With the increasing pressure, the indirect band-gap became smaller than the direct band-gap. Based on the V-P results, the band-gap of the monolayer MoS_2_ initially increased from 1.83 eV to a maximum value around 2 eV at 2 GPa pressure. It then decreased with the increase in applied pressures above 2 GPa. Moreover, the critical hydrostatic pressure around 2 GPa of the MoS_2_ band-gap for the direct–indirect transition of the band structure is very much consistent with the literature, reported as 1.9 GHz from the experimental data [32]. Consistent values of the band-gaps for the monolayer MoS_2_ were obtained at each pressure from 2 to 8 GPa based on V-P, V-H, and C-H, as depicted in Figure 4a.

The band-gap values of the monolayer semiconductors of Sb and BP consistently decreased linearly with the increase in applied hydrostatic pressure. The band-gap values of BP decreased with the increase in hydrostatic pressures from 0 to 8 GPa, as shown in Figure 4b. The decrease slope is the same for the V-P, V-H, and C-H functionals. Moreover, a similar tendency was also found for the relationship between the band-gap and pressure for the monolayer Sb, as shown in Figure 4c. The critical pressures, denoted as Pcr-bg0, where the band-gaps reduced to zero are reported in Table 3. It is noticed that the average values of the critical pressures are 4 GPa for the monolayer Sb and 6.5 GPa for the monolayer BP.

Therefore, the monolayer Sb can be deeply tuned by applying much lower pressure, as compared to the monolayer BP and MoS_2_. Specifically, the band-gap of Sb can be tuned to zero at low pressure around 3 GPa, estimated from the average value, which is experimentally achievable for the nanostructure of the monolayer Sb.

The bulk MoS_2_ was reported by applying high pressure up to 40 GPa so that the 0.4 eV band-gap was reached from the first-principle calculations [42]. This can be explained in that the nanostructure of MoS_2_ is a compound semiconductor with much stiffer mechanical properties, as shown in Figure 4a,d. Therefore, it requires much higher pressure for deformation to tune its band-gap. This phenomenon can be explained from the energy-volume curves that are well developed in the literature [43,44].

**Figure 5:**
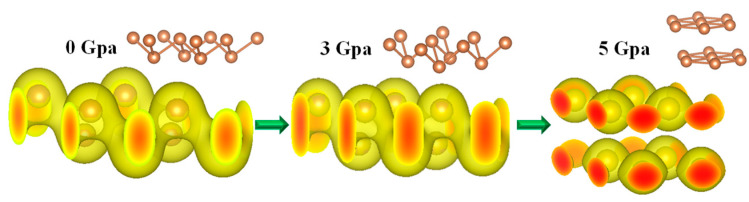
Charge densities of the monolayer Sb under pressures of 0 GPa, 3 GPa, and 5 GPa; phase transformation under the critical pressure around 5 GPa, where the monolayer transforms to a double-layer nanostructure.

In contrast, we investigated the lattice displacements of the monolayer MoS_2_, BP, and Sb for investigating the dynamical stability of the nanostructures. It is clearly shown that before critical pressure, the in-plane lattice parameters of a and b, as well as the out-of-plan lattice parameter d, were all stable with small displacements. However, after a critical pressure was reached, the in-plane lattice parameters of a changed relatively significantly and the out-of-plan lattice parameter d remained stable for the monolayer BP. Instead, after critical pressure of approximately 3 GPa, the out-of-plan lattice parameter d and the in-plane lattice parameters of a and b significantly changed for the monolayer Sb. Combined with analysis from Figure 5, we believe that the monolayer Sb became unstable and transformed from a semiconductor to a metal above the critical pressure of approximately 3 GPa.

Further investigation of the charge densities showed that the monolayer Sb can be completely transformed from a semiconductor to a metal. The 3D diagrams of the charge density distribution of the monolayer Sb under different pressures were prepared using VESTA, as shown in Figure 5. It is clearly depicted that under different pressures, the distributions of the charge density significantly changed. When the pressure was relatively low, a shared electron pair, i.e., a covalent bond, was formed between two Sb atoms. When the hydrostatic pressure was applied above the critical pressure up to 5 GPa, the covalent bond was completely broken, and the shared electron pair was not formed. Moreover, with the increasing pressure, the electron clouds of the shared electron pairs were squeezed, and the shared area became smaller. Finally, the shared electron pairs were destroyed, indicating a phase transition. Therefore, the crystal nanostructure of the monolayer Sb material correspondingly changed from a monolayer to a bilayer structure with different lattice parameters so that the metal property similar to bulk Sb was reassembled in nature. Therefore, the reassembled metallic characteristics from the single-layer semiconductor Sb was estimated at a critical pressure ranging from 3 to 5 GPa or 4 GPa on average from our calculations. This novel property opens a new opportunity for the band-gap engineering and tunability of monolayer Sb and BP nanomaterial applications to nanodevices and nanosensors by applying pressure.

## 4. Conclusions

In summary, the band-gap characteristics of the two-dimensional monolayer semiconductors of Sb, BP, and MoS_2_ were intensively investigated by applying different pressures from 0 to 8 GPa for case studies, based on the first-principle calculations of VASP-GGA-PBE, VASP-GGA-HSE06, and CASTEP-GGA-HSE06 functionals in this paper. This benchmark study indicates that the monolayer Sb exhibits semiconductor properties with an indirect band-gap and an estimated value of 1.77 eV. Moreover, the band-gap values of the monolayer semiconductors of Sb and BP decreased linearly with the increase in the applied hydrostatic pressures. However, the band-gap values of the monolayer MoS_2_ did not change monotonically with the applied pressures. A critical hydrostatic pressure around 2 GPa for the direct–indirect transition of the band structure of the monolayer MoS_2_ is very much consistent with the literature, reported as 1.9 GHz from the experiment data [34].

Further investigation of the charge densities showed that the monolayer Sb can be completely transformed from a semiconductor to a metal at a critical pressure ranging from 3 to 5 GPa or 4 GPa, as estimated from our calculations. Therefore, the monolayer Sb can be deeply tuned by applying a much lower pressure, compared to BP and MoS_2_. Specifically, the band-gap of Sb can be tuned to zero at a low pressure around 4 GPa, as estimated from our calculations, which is experimentally achievable for the nanostructure of the monolayer Sb. The band-gap tunability of the monolayer semiconductors of Sb, BP, and MoS2 under hydrostatic pressure opens new opportunities for potential applications to nanoelectronic, flexible electronics and optoelectronic devices, as well as sensors.

## Figures and Tables

**Table 1:** List of band-gap values obtained by using different Hamiltonians; * represents the data from [24].

Hamiltonian	Band-Gap (eV)
VASP-LDA	1.21
VASP-GGA-PBE	1.27
VASP-GGA-HSE06	1.87
CASTEP-GGA-HSE06	2.26
CASTEP-GGA-HSE06 *	2.28

**Table 2:** List of band-gap values by using different methods.

Monolayer	BP	MoS_2_	Sb
**VASP-PBE (V-P)**	0.89	1.83	1.27
**CASTEP-HSE06 (C-H)**	1.84	2.14	2.28
**Average V-P/C-H**	1.37	1.98	1.78
**EXP Data**	**1.45 [18]**	**1.90 [41]**	**NA**
**Deviation (%) of Average V-P/C-H**	**−5.59**	**4.26**	**NA**
**VASP-HSE06 (V-H)**	1.65	2.01	1.87
**Deviation (%) of V-H**	**13.79**	**5.79**	**NA**

EXP: experimental.

**Table 3:** List of the critical pressures, P_cr-__bg0_, by using different methods.

P_cr-bg0_	V-P	C-H	Average V-P/C-H
BP	4.0 GPa	9.0 GPa	6.5 GPa
Sb	3.0 GPa	5.0 GPa	4.0 GPa
